# The little things that matter: how bioprospecting microbial biodiversity can build towards the realization of United Nations Sustainable Development Goals

**DOI:** 10.1038/s44185-022-00006-y

**Published:** 2022-12-07

**Authors:** Paton Vuong, Sandy Chong, Parwinder Kaur

**Affiliations:** 1 UWA School of Agriculture & Environment, University of Western Australia, Perth, Australia; 2 Faculty of Science & Engineering, Curtin University, Perth, Australia; 3 United Nations Association of Australia (WA Division), Perth, Australia

**Keywords:** Biotechnology, Microbiology

## Abstract

The astronomical number of individual microorganisms that exist on Earth provides an immeasurable trove from which potential microbial-based solutions can be drawn upon to drive the development of sustainable industries. However, there is little information documenting the spectrum of global microbial biodiversity and how human activity has impacted the taxonomic and functional diversity of microbial communities. Here, we discuss how promoting microbial innovation can encourage environmental, social, and corporate governance investments towards protecting global biodiversity for all life whilst meeting the 2030 United Nations Sustainable Development Goals.

Biodiversity drives ecological and environmental processes, so its decline across the globe threatens the vital ecosystem services that all life relies upon^1^. The conservation of biodiversity is critical because ecosystems, the taxa therein, and their associated genetic information are key contributors to sustainable development^2^. As such, the preservation of global biodiversity is required for achieving United Nations Sustainable Development Goals (UN SDGs) that seek to improve the livelihoods of all people of the world through the development of sustainable industries and the promotion of sustainable living (https://sdgs.un.org/). Although there have been plenty of studies documenting the biodiversity loss of plants and animals, the effects of human activity on global microbial biodiversity remain virtually unknown^3^. This knowledge gap needs to be remedied as the global microbiome is core to the ecosystem processes that support all lifeforms, with changes in microbial responses providing a key indicator of global health^4^. In this paper, we highlight how understanding microbial biodiversity can help drive conservation efforts, and how microbial-based innovations developed by bioprospecting microbial life can encourage investments from environmental, social, and corporate governance (ESG) initiatives toward the realization of the UN SDGs (Fig. 1).

**Fig. 1:**
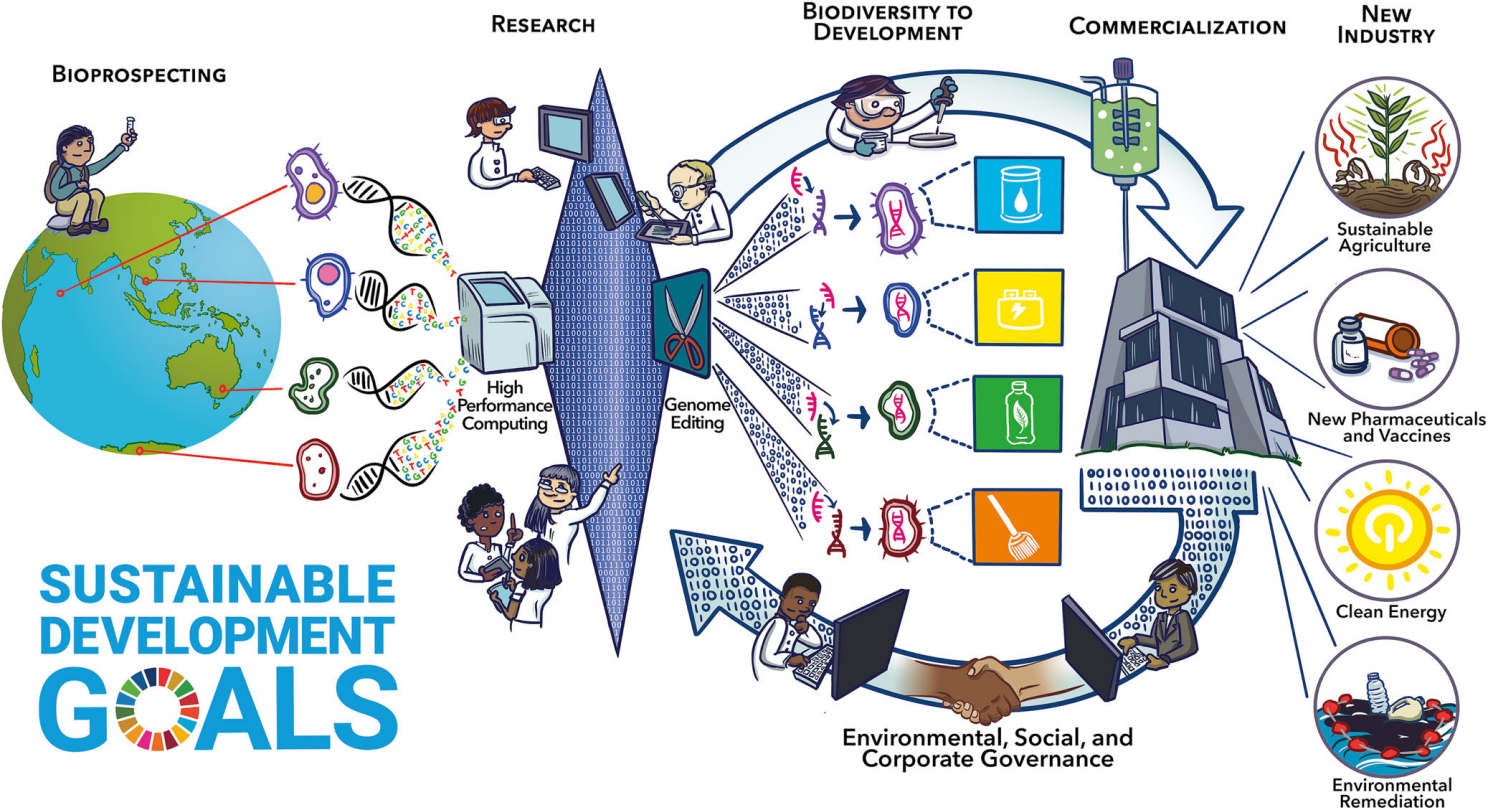
A schematic visualization of the potential of microbial bioprospecting for driving forward the development of sustainable solutions. Credit: Adam Fotos.

## Understanding the unseen microbial diversity within ecosystems

Microbial biodiversity is unperceivably immense, with an estimated ~10^12^ microbial species present on Earth, with bacterial and archaeal microorganisms alone comprising ~10^30^ cells^5^. This means that there are more prokaryotic cells on Earth than stars estimated to exist in the observable universe (10^22^ to 10^24^) by the European Space Agency (https://www.esa.int/). Along with the volumes of microorganisms that need to be accounted for, the difficulty in documenting global microbial diversity is that there are no straightforward automatic or remote monitoring systems that can be relied upon for the quantification of microbial communities. Instead, such efforts are reliant on global databases that collate microbial sequence data from numerous individual studies, or from large-scale collaborative efforts which then need to undergo extensive computational analysis in order to provide the information needed for microbiome comparisons^6–8^. Although these large-scale sequencing studies have made a lot of headway into understanding the global distribution of microbial diversity, performing frequent repeated surveys is challenging due to the analytical and procedural constraints. As a result, our understanding of the spatial-temporal dynamics of microbial community structures remains limited.

Climate change and environmental perturbations due to human activity are a key driver of plant and animal extinctions, but conservation efforts often overlook microbial populations when addressing the loss of biodiversity^4^. Predictive modelling of soil community structures has indicated that warming temperatures due to climate change are likely to result in a mass homogenization of global soil microbial communities, leading to an overall loss of microbial biodiversity^6^. Microorganisms play essential roles in biogeochemical processes, and the disturbance of these microbial systems on a global scale may result in dramatic ecological issues, such as disruptions of food webs due to nutrient cycling changes, and increased greenhouse gas production due to alterations of the carbon cycle. As these ramifications can lead to further biodiversity loss up the ecological chain, more studies are required to understand the nuances of global environmental microbial ecology, and how changes in microbial community structures affect ecosystem functions.

Large-scale sequencing studies have been pivotal in understanding global microbial biodiversity, with the additional benefit of including genetic information that can be used to further biotechnological studies. Microbiomes and the microorganisms therein can perform a vast number of biochemical processes with the potential to drive sustainable innovations through the development of microbial-based bioeconomies, necessitating the inclusion of microbiology into the UN SDGs^9^. Advances in technology have generated the massive amounts of sequence data that are now commonplace, shifting microbiology towards the realm of data science and allowing researchers access to unparalleled discoveries by delving into the world of big data^10^. This has created a rich cache of information on microbial biodiversity for bioprospection, a process that searches for (micro)organisms holding the genetic information for bioprocesses or bioproducts of commercial or industrial interest. Environmental sequence data from different ecosystems can be “bioprospected” for microbial functions that can be exploited for use in developing sustainable microbial-based innovations with the potential to replace current economic models and industrial processes that rely on fossil fuels and their derivatives^11^.

## How exploring microbial biodiversity can drive sustainable development

Advances in microbial ecology and biotechnology show promise in providing innovative and alternative methods toward established linear economic models. Table 1 provides an overview of how bioprospecting and microbial-based solutions delivered within the scope of ESGs could help further sustainable industries and aid in meeting the UN SDGs. Prime examples include: (i) bioprospecting genomes for new biosynthetic pathways that provide access to novel routes of drug production^12^; (ii) microorganisms that can convert agro-industrial waste into added-value products, such as biofuels, biochemicals and bio-based materials can help promote a green economy by lessening the dependency on fossil fuels^13^; (iii) or microalgae that can sequester CO_2_ to provide biomass for bioproduct production, as well as being potential sources of landless food production, thus reducing the amount of land use needed for agriculture^14^. These and other microbial-based solutions are sustainable approaches that either improve the wellbeing of humans, or address some of the longstanding environmental issues that are major contributors to global biodiversity loss.

**Table 1:** Microbial-based industries and the UN Sustainable Development Goals.

UN sustainable development goals	Microbial-based bioeconomy facilitating ESG outcomes	Microbial-based solutions - examples	Improvements through microbial bioprospecting
Sustainable Innovations for Industry and Economic Development			
SDG 1: No Poverty SDG 8: Decent Work and Economic Growth	The creation of microbial-based industries that meet the demand for sustainable practices and products is key for building new bioeconomic opportunities.	Microbial biorefineries that can produce bio-chemical and bio-fuel alternatives from renewable resources to replace fossil fuel derivatives^30^.	Bioprospecting of extreme environments can yield robust microbes with bioproducts that can tolerate a broad range of operating conditions present across various industrial applications^11^.
SDG 7: Affordable and Clean Energy SDG 9: Industry, Innovation and Infrastructure SDG 12: Responsible Consumption and Production	A microbial-based bioeconomy promotes industrialization with consideration of the environmental impacts of the utilization, production, and consumption of materials.	Utilization of agro-industrial waste residues such as lignin via microbial-based conversion into value-added bio-based fuels, chemicals and materials^13^.	Discovering novel strains with improved ligninolytic capability or more efficient lignin depolymerization that can compete with or approve upon current chemo-catalytic methods^13^.
SDG 13: Climate Action	Use of sustainable alternatives to fossil fuels and their derivatives for production materials resulting in smaller or carbon-negative footprints.	Microbial biocatalysts that can convert sequestered CO_2_ into usable bioproducts^30^.	Identification of suitable microorganisms with efficient and diverse substrate and feedstock usage that can utilize various renewable resources for metabolizing compounds of interest^11^.
SDG 14: Life Below Water	Production of plastic alternatives that are safer for life in marine environments.	Microbially-produced bioplastics are a promising bio-based, biodegradable alternative that can use renewable resources as feedstock for biopolymer production^31^.	Discovery of novel strains and metabolic pathways can improve all aspects of microbial bioplastic production, such as diversifying feedstock/substrate usage, more efficient production/extraction processes and improving end-of-life disposal methods^31^.
Responsible Food Production and Arable Land Use			
SDG 2: Zero Hunger SDG 15: Life on Land	Microbiology has the potential to provide sustainable agricultural solutions through efficient land use and improving soil health.	Introduction of beneficial microorganisms into soil microbial communities to increase crop yield and reduce fertilizer use as an avenue towards sustainable agriculture^32^. Microalgae farming can provide either food or biomass for use in other sustainable industries and requires less arable land than other sources of renewable production^14^.	Bioprospecting and cataloguing microorganisms that are acclimated to specific ecological conditions for better rates of establishment in soil^32^.
Disease Monitoring and Provision of Good Health			
SDG 3: Good Health and Well-Being SDG 6: Clean Water and Sanitation	Environmental microbial community profiles can be either monitored for pathogens or searched for drug production pathways. This is crucial for the prevention of pandemics, as well as providing new sources for pharmaceutical development.	Microorganisms are the main sources of drug discovery and production and the search for new antibiotics is the key to combatting anti-microbial resistant pathogens^12^. Metagenomic screening as a surveillance system where data on outbreaks or potentially high-risk microbes detected in different settings, such as food, water or other environmental sources are shared across a global network^33^.	Mining genomes can unveil new biosynthetic pathways that present potential avenues to novel drug production^12^.
Sharing of Transformative and Technical Knowledge			
SDG 4: Quality Education SDG 17: Partnerships for the Goals	Collaborative efforts that share valuable data help develop diverse industries and promote sustainable practices within a microbial-based bioeconomy.	Sequence databases such as the International Nucleotide Sequence Database Collaboration (https://www.insdc.org/), along with reference databases for proteins and biosynthetic gene clusters provide knowledge that can be used to further understanding in microbial ecology and biotechnology.	Publicly available data, along with accessible metadata provides is the backbone of a knowledge-based bioeconomy by providing the “big data” needed for large-scale bioprospecting efforts.

There are still many challenges that need to be addressed before the widespread adoption of microbial-based solutions can occur. Inefficiencies within the production processes, such as substrate and product inhibition, inconsistent fermentation rates, challenges with contamination, and issues with product purification and yield are often what prevent microbial production of added-value products from mass commercialization^15^. Bioprospecting of metagenomic data from environmental microbiomes has been one of the keys to discovering novel genes and is one of the first major steps into addressing the issues faced in microbial biotechnology^11^. As such, bioprospecting of microbial biodiversity is the vital contributor that reconciles nature-based solutions with synthetic biology through the discovery of metabolic potential that can be used to enhance current microbial bioprocesses for use in sustainable industry.

This leads to one of the most important questions regarding microbial bioprospecting, “where is the best place to search?” In terrestrial microbiomes, areas with high aboveground biodiversity often contain low diversity soil microbial communities, and this lack of correlation may make currently defined biodiversity hotspots unsuitable reference points for the preservation of microbial diversity^16^. On the contrary, extreme environments that are often deemed hazardous to life can harbor high microbial diversity that could provide a robust supply of bioproducts usable under a broad range of physicochemical conditions^17^. Even the urban microbiome, an unconventional setting for biodiversity studies, has recently been shown to contain many novel bacterial species and CRISPR arrays^18^. Taking these points into consideration, perhaps the answer for the best ecosystems for bioprospecting is “anywhere where it’s needed”.

Any region that is undergoing ecological transition, such as areas of urban or agricultural expansion, or undergoing ecosystem restoration, should be a target for microbial surveys. Microorganisms have always been overlooked compared to plants and animals^3^, but as they have been shown to be vital ecosystem providers^4^, microbial communities should be included in biodiversity measurements when addressing biodiversity targets in development or restoration projects^19^. In cases like these, it can be argued that the bioprospecting aspect provided by microbial surveys is an added-value product from conservation efforts. Microbial-based innovations derived from understanding microbial biodiversity provide a potential avenue of reconciliation to the longstanding conflict between the need to protect biodiversity against the demand for economic progress, by building wealth through sustainable development^20^.

## How a wealth of microbial information can fuel conservation efforts

The Aichi Biodiversity Target was set forth in 2010 as an international collaborative through the Convention on Biological Diversity to address the rapid decline in biodiversity observed across the globe by 2020 (https://www.cbd.int/sp/targets/). Unfortunately, even with global cooperation, none of the goals set forth were able to be fully realized at the planned 2020 end date. Two of the major challenges encountered by the Aichi Targets were insufficient funding, and science-policy knowledge gaps during decision making^21^. The difficulties in meeting the Aichi Targets lies in the inherent conflict between the demand for economic growth and the need to prevent biodiversity loss, and more engagement with the socioeconomic aspects involved is required to drive better transformative policies and encourage sustainable development^22^. To address these deficiencies in the future, a better understanding of global microbial biodiversity and how it can drive microbial-based solutions may help promote ESG investments into this area.

The integration of microbial diversity into conservation policies requires changes into how conservation efforts are directed. The approach where areas of high species richness are deemed as priorities for protection as “biodiversity hotspots” typically only account for vertebrate and plant life, and this method often overlooks the multitude of areas where ecological transition occurs, in which active losses in species are occurring^23^. Protected areas in terrestrial ecosystems often do not consider the soil microbiome within their policies or management plans, and in some cases, these areas confer no benefits to the soil microbial populations present^24^. More engagement towards the understanding of microbial ecology is needed to improve environmental management decisions to encompass ecosystems in their entirety, and to better direct attention to ecological areas that are overshadowed through anachronistic measures of biodiversity.

Studies in microbial ecology contribute vital knowledge towards the conservation of biodiversity, as a healthy microbiome has been shown to act as a steward that bolsters the resiliency of both wildlife and ecosystems against biodiversity loss^25^. This is especially vital in areas of ecological transition, where disturbances of the microbiome correlate with deleterious effects on animal, human, and plant health. By encouraging microbial surveys in regions where ecological interactions have been underexplored or where massive shifts are expected to occur, we can get a better grasp of the status of global microbial populations, as well as support the growth of microbial-based biotechnology. This encourages a positive loop between bioprospecting and the conservation of biodiversity, whereby understanding and preventing biodiversity loss also gives rise to more taxa to prospect, which further provides opportunities to discover microbial-based answers to sustainable solutions.

As shown in Table 1, microbial-based innovations have demonstrated the potential in driving sustainable development, alleviating the conflict between economic progress and biodiversity conservation. Therefore, the preservation of microbial biodiversity should be promoted as tantamount to investing in securing future sources of productivity. The marine microbiome, for example, was found to host thousands of new biosynthetic gene clusters^7^ which are valuable genetic targets for drug development^12^. However, the long-term response from perturbations due climate change and human activity from the marine microbial populations remain largely unknown^4^. Microbial bioprospecting provides a potential bridge that can link sustainable development and conservation through the demonstration of the value of biodiversity and how it can contribute to transformable industries. The biological discoveries can attract investments from synthetic biology and/or biotechnology companies involved in microbial bio-engineering, with Gingko Bioworks (https://www.ginkgobioworks.com/) and Amyris (https://amyris.com/) being prominent examples within this lucrative multi-million dollar industry. This provides a springboard to integrate microbial ecology into the decision-making processes within transformable governance, providing more informed policies that can better address the post-2020 Aichi Targets as well as the UN SDGs^26^.

Understanding the global microbiome requires harnessing data on a massive scale, and will involve large collaborative efforts that require expertise from many interdisciplinary fields and institutions to address the broad and technical issues encountered during the research process^27^. Biodiversity is often understudied in the developing world and is it critical that we ensure that the scientists, innovators, and traditional knowledge holders from these countries are appropriately engaged and involved in the research and decision-making processes^28^. We also need to ensure that any genetic discoveries found during bioprospecting are properly attributed to the regions where they are found. Although the Nagoya Protocol covers the access, equitable-use, and benefit-sharing of genetic information, this becomes fuzzy when applied to microorganisms^29^. Microorganisms and their associated functions are often ubiquitous due to sequence homology and orthologs, which potentially leads to issues when discoveries are made independently from different sampling sites. The concerns are that genomic discoveries could be searched and attributed to microorganisms containing homologous biology from regions with less stringent regulations on data access and dissemination. For situations like these, we are reliant on the corporate social responsibility of institutions and organisations to properly contribute discoveries for the common good.

## Towards a biodiverse and sustainable future

Incentives that drive ESG investments into sustainable projects and the protection of biodiversity forms the foundation for the development of successful sustainable industries and practices. Through bioprospecting sequence data obtained from global ecosystems, the discovery of novel microbial candidates and bioprocesses adds to our understanding of microbial ecology. This engagement drives transformative governance, encourages the protection of biodiversity, and fuels improvements within microbial biotechnology. The sharing of knowledge and resources is fundamental in sustainability, as it ensures that nations of all standings have the capability to achieve responsible industrialization, promotes cooperation between all members, and provides informed decision-making towards conservation efforts. The proposed applications of microbial-based solutions for current linear economic models can pave the way for the realization of the UN SDGs and the prevention of the decline of global biodiversity.

## Data Availability

All data generated or analyzed during this study are included in this published article.

## Code availability

No custom code or mathematical algorithms were associated with this work.

## References

[1] Cardinale, B. J. et al. Biodiversity loss and its impact on humanity. *Nature* **486**, 59–67 (2012). 22678280 10.1038/nature11148

[2] Blicharska, M. et al. Biodiversity’s contributions to sustainable development. *Nat. Sustain.* **2**, 1083–1093 (2019). 10.1038/s41893-019-0417-9

[3] Thaler, D. S. Is global microbial biodiversity increasing, decreasing, or staying the same. *Front. Ecol. Evol.* **9**, 221 (2021). 10.3389/fevo.2021.565649

[4] Cavicchioli, R. et al. Scientists’ warning to humanity: microorganisms and climate change. *Nat. Rev. Microbiol.* **17**, 569–586 (2019). 31213707 10.1038/s41579-019-0222-5 PMC7136171

[5] Locey, K. J. & Lennon, J. T. Scaling laws predict global microbial diversity. *Pro. Natl. Acad. Sci. USA* **113**, 5970–5975 (2016). 10.1073/pnas.1521291113 PMC4889364 27140646

[6] Guerra, C. A. et al. Global projections of the soil microbiome in the Anthropocene. *Global Ecol. Biogeogr.* **30**, 987–999 (2021). 10.1111/geb.13273 PMC7610617 33867861

[7] Paoli, L. et al. Biosynthetic potential of the global ocean microbiome. *Nature* **607**, 111–118 (2022). 35732736 10.1038/s41586-022-04862-3 PMC9259500

[8] Bahram, M. et al. Structure and function of the global topsoil microbiome. *Nature* **560**, 233–237 (2018). 30069051 10.1038/s41586-018-0386-6

[9] D’Hondt, K. et al. Microbiome innovations for a sustainable future. *Nat. Microbiol.* **6**, 138–142 (2021). 33510435 10.1038/s41564-020-00857-w

[10] Eren, A. M. et al. Community-led, integrated, reproducible multi-omics with anvi’o. *Nat. Microbiol.* **6**, 3–6 (2021). 33349678 10.1038/s41564-020-00834-3 PMC8116326

[11] Krüger, A., Schäfers, C., Busch, P. & Antranikian, G. Digitalization in microbiology - paving the path to sustainable circular bioeconomy. *New Biotechnol.* **59**, 88–96 (2020). 10.1016/j.nbt.2020.06.004 32750680

[12] Hemmerling, F. & Piel, J. Strategies to access biosynthetic novelty in bacterial genomes for drug discovery. *Nat. Rev. Drug Discov.* **21**, 359–378 (2022). 35296832 10.1038/s41573-022-00414-6

[13] Goncalves, C. C. et al. Bioprospecting microbial diversity for lignin valorization: dry and wet screening methods. *Front. Microbiol.* **11**, 1318 (2020). 32582068 10.3389/fmicb.2020.01081 PMC7295907

[14] Ullmann, J. & Grimm, D. Algae and their potential for a future bioeconomy, landless food production, and the socio-economic impact of an algae industry. *Org. Agric.* **11**, 261–267 (2021). 10.1007/s13165-020-00337-9

[15] Leonov, P. S., Flores-Alsina, X., Gernaey, K. V. & Sternberg, C. Microbial biofilms in biorefinery - towards a sustainable production of low-value bulk chemicals and fuels. *Biotechnol. Adv.* **50**, 107766 (2021). 33965529 10.1016/j.biotechadv.2021.107766

[16] Cameron, E. K. et al. Global mismatches in aboveground and belowground biodiversity. *Conserv. Biol.* **33**, 1187–1192 (2019). 30868645 10.1111/cobi.13311

[17] Becker, J. & Wittmann, C. Microbial production of extremolytes - high-value active ingredients for nutrition, health care, and well-being. *Curr. Opin. Biotechnol.* **65**, 118–128 (2020). 32199140 10.1016/j.copbio.2020.02.010

[18] Danko, D. A global metagenomic map of urban microbiomes and antimicrobial resistance. *Cell* **184**, 3376-3393.e3317, 10.1016/j.cell.2021.05.002 (2021). 10.1016/j.cell.2021.05.002 PMC8238498 34043940

[19] Simmonds, J. S. Moving from biodiversity offsets to a target-based approach for ecological compensation. *Conserv. Lett.* **13**, e12695, 10.1111/conl.12695 (2020).

[20] Czech, B. Prospects for reconciling the conflict between economic growth and biodiversity conservation with technological progress. *Conserv. Biol.* **22**, 1389–1398 (2008). 19076872 10.1111/j.1523-1739.2008.01089.x

[21] Xu, H. et al. Ensuring effective implementation of the post-2020 global biodiversity targets. *Nat. Ecol. Evol.* **5**, 411–418 (2021). 33495589 10.1038/s41559-020-01375-y

[22] Otero, I. et al. Biodiversity policy beyond economic growth. *Conserv. Lett.* **13**, e12713 (2020). 32999687 10.1111/conl.12713 PMC7507775

[23] Marchese, C. Biodiversity hotspots: a shortcut for a more complicated concept. *Global Ecol. Conserv.* **3**, 297–309 (2015). 10.1016/j.gecco.2014.12.008

[24] Zeiss, R. et al. Challenges of and opportunities for protecting European soil biodiversity. *Conserv. Biol.* **36**, e13930 (2022). 35510330 10.1111/cobi.13930

[25] Peixoto, R. S. et al. Harnessing the microbiome to prevent global biodiversity loss. *Nat. Microbiol.* **7**, 1726–1735 (2022). 35864220 10.1038/s41564-022-01173-1

[26] Visseren-Hamakers, I. J. et al. Transformative governance of biodiversity: insights for sustainable development. *Curr. Opin. Environ. Sustain.* **53**, 20–28 (2021). 10.1016/j.cosust.2021.06.002

[27] Börner, K., Silva, F. N. & Milojević, S. Visualizing big science projects. *Nat. Rev. Phys.* **3**, 753–761 (2021). 10.1038/s42254-021-00374-7

[28] Asase, A., Mzumara-Gawa, T. I., Owino, J. O., Peterson, A. T. & Saupe, E. Replacing “parachute science” with “global science” in ecology and conservation biology. *Conserv. Sci. Pract.* **4**, e517 (2022).

[29] Pascual, J., Tanner, K., Vilanova, C., Porcar, M. & Delgado, A. The microbial terroir: open questions on the Nagoya protocol applied to microbial resources. *Microb. Biotechnol.* **14**, 1878–1880 (2021). 34311495 10.1111/1751-7915.13839 PMC8449654

[30] Liu, Z., Wang, K., Chen, Y., Tan, T. & Nielsen, J. Third-generation biorefineries as the means to produce fuels and chemicals from CO2. *Nat. Catal.* **3**, 274–288 (2020). 10.1038/s41929-019-0421-5

[31] Rosenboom, J.-G., Langer, R. & Traverso, G. Bioplastics for a circular economy. *Nat. Rev. Mater.* **7**, 117–137 (2022). 35075395 10.1038/s41578-021-00407-8 PMC8771173

[32] Diaz-Rodriguez, A. M. et al. The current and future role of microbial culture collections in food security worldwide. *Front. Sustain. Food Syst.* **4**, 101 (2021). 10.3389/fsufs.2020.614739

[33] Ko, K. K. K., Chng, K. R. & Nagarajan, N. Metagenomics-enabled microbial surveillance. *Nat. Microbiol.* **7**, 486–496 (2022). 35365786 10.1038/s41564-022-01089-w

